# Generalization of the modulatory effect of social interaction on personal space

**DOI:** 10.3389/fpsyg.2023.1148395

**Published:** 2023-06-15

**Authors:** Shulei Cui, Tianshu Yang, Ning Liu

**Affiliations:** ^1^State Key Laboratory of Brain and Cognitive Science, Institute of Biophysics, Chinese Academy of Sciences, Beijing, China; ^2^College of Life Sciences, University of Chinese Academy of Sciences, Beijing, China; ^3^Institute of Artificial Intelligence, Hefei Comprehensive National Science Center, Hefei, China

**Keywords:** social interaction, personal space, generalization, modulatory effect, healthy participants

## Abstract

**Introduction:**

Personal space (PS) is a safe area around an individual’s body that affects spatial distance when socially interacting with others. Previous studies have shown that social interaction may modulate PS. However, these findings are often confounded by the effects of familiarization. Furthermore, whether the potential regulatory effects of social interaction on PS can be generalized from interacting confederates to strangers remains unclear.

**Methods:**

To answer these questions, we enrolled 115 participants in a carefully designed experiment.

**Results:**

We found that prosocial interaction in the form of a cooperative task effectively reduced PS, and this regulatory effect could be generalized from interacting confederates to non-interacting confederates.

**Discussion:**

These findings deepen our understanding of PS regulation and may be aid in the diagnosis and rehabilitation of dysfunctional social behaviors.

## 1. Introduction

Personal space (PS) is a safe zone around the body where intrusion by others may cause discomfort (Hayduk, 1978, 1983). During social interactions, we automatically monitor and regulate this space to maintain an appropriate interaction distance from others (Sommer, 1959; Horowitz et al., 1964; Hall, 1966). For example, PS tends to be reduced during social activities to promote affiliation (Kahn and McGaughey, 1977), but enlarged when facing threatening (Dosey and Meisels, 1969; Hayduk, 1978) or untrustworthy people (Rosenberger et al., 2020; Massaccesi et al., 2021). Therefore, PS plays a critical role in social communication. Unsurprisingly, various disorders involving dysfunctional social behavior can influence PS. Previous studies have reported enlarged or inflexible PS in patients with schizophrenia (Horowitz et al., 1964; Park et al., 2009; Holt et al., 2015). For example, children with autism spectrum disorder (ASD) exhibit greater PS requirements than children with typical development (Gessaroli et al., 2013; Candini et al., 2017), although several studies have shown the opposite results in adults with ASD (Kennedy and Adolphs, 2014; Asada et al., 2016). In addition, patients with bilateral damage to the amygdala, a key region related to emotional and social perception, show a substantially reduced PS compared to healthy subjects (Kennedy et al., 2009).

Previous studies have demonstrated that PS can be modulated by situational, social, and psychological factors, as well as individual characteristics such as sex, age, cultural norms, infant-caregiver attachment, perception of morality, and familiarity between interacting parties (Little, 1965; Willis, 1966; Hayduk, 1978; Bar-Haim et al., 2002; Cristani et al., 2011; Iachini et al., 2015, 2016; Pellencin et al., 2017). For example, people tend to expand their PS when approached by someone described as immoral (Iachini et al., 2015), but reduce their PS for more intimate social relationships (Little, 1965; Willis, 1966; Cristani et al., 2011). Recent studies have also suggested that PS can be modulated by social interaction (Gessaroli et al., 2013; Candini et al., 2017, 2019; Patané et al., 2017; Rosenberger et al., 2020; Massaccesi et al., 2021). For example, Gessaroli et al. (2013) reported that typically developed children will reduce their PS after reading an illustrated book with a female confederate. This phenomenon has also been reported in healthy adults following the cooperative use of tools (Patané et al., 2017). Although the modulatory effects of social interaction on PS has rarely been reported in individuals with social interaction deficits, such as ASD (Gessaroli et al., 2013; Candini et al., 2017, 2019), social interaction can modulate PS in children with low social impairment ASD under more interactive tasks [e.g., playing together with Lego blocks (Candini et al., 2017) versus reading together (Gessaroli et al., 2013)]. These studies suggest that social interaction should be considered in the diagnosis and rehabilitation of disorders with dysfunctional social behavior. Indeed, peer-mediated intervention shows promise as a treatment for various disorders with social behavior deficits (e.g., ASD) (Chang and Locke, 2016).

Although there is evidence that social interaction modulates PS, it is unknown whether this effect is simply due to confederate familiarization as opposed to social interaction itself. A previous study conducted a control experiment to limit the effects of confederate familiarity by including a condition in which the confederate was in the same room as the participant but did not interact, in addition to the condition under which the confederate and participant read a book together (Gessaroli et al., 2013). Obviously, such a control experiment resulted in different levels of confederate familiarization rather than completely excluding the impact of familiarization. Note that PS can be modulated by familiarity between interacting parties (Little, 1965; Willis, 1966; Cristani et al., 2011). On the other hand, in previous studies, PS was measured before and after social interaction with the same interacting confederate (Gessaroli et al., 2013; Candini et al., 2017, 2019; Patané et al., 2017; Massaccesi et al., 2021). Therefore, whether the modulatory effect of social interaction on PS, if it exists, can be generalized from cooperative confederates to strangers remains unresolved. Answering these questions is essential for assessing the potential of social interaction to improve PS in patients with dysfunctional social behavior.

In this study, we aimed to answer the above questions by increasing the number of confederates and comparing PS with cooperative confederates and non-cooperative confederates before and after social interaction.

## 2. Materials and methods

### 2.1. Participants

The sample size estimation (*n* = 98, based on the desired power = 0.80, alpha level = 0.05, effect size = 0.25) resulted from an *a priori* power calculation using G*Power 3.1.9.7 based on the most comparable study by Rosenberger et al. (2020). A total of 120 healthy adult Chinese participants (60 males and 60 females) were recruited for this study. Five participants were excluded from further analysis because their PS was outside the range of mean ± 3 standard deviations (SD) across all participants, or they did not complete the experimental procedure. That is, the sample size for the final data analysis in the present study was 115 participants (57 males and 58 females, 23.37 ± 2.53 years). Participants were randomly assigned to either the Cooperative group (23.76 ± 2.74 years) or Control group (22.98 ± 2.26 years) (see Table 1). Four additional participants [two females (f1 and f2) and two males (m1 and m2), 22.00 ± 1.73 years] were recruited as confederates. Images of their faces were taken and used as stimuli. Due to personal reasons, the original m2 confederate was unable to participate in the experiments after cooperation with one participant, and thus a new male confederate (m2) was recruited to participate in the remaining experiments. Images of the first m2 were used for 38 participants and images of the second m2 were used for the remaining 77 participants.

**TABLE 1:** Demographics of 115 participants.

		N	Age range (Years)	Age (Years)	AQ	STAI-State	STAI-Trait	Actual accompanied (%)	Expected accompanied (%)
Control	Male	28	20∼28	22.96 ± 1.82	21.43 ± 5.48	34.14 ± 10.35	39.46 ± 9.77	59.11 ± 23.22	49.82 ± 19.27
	Female	29	19∼30	23.00 ± 2.66	21.17 ± 6.22	33.97 ± 8.63	38.38 ± 9.05	54.14 ± 25.29	46.90 ± 19.11
	All	57	19∼30	22.98 ± 2.26	21.30 ± 5.82	34.05 ± 9.43	38.91 ± 9.34	56.58 ± 24.20	48.33 ± 19.07
Cooperative	Male	29	18∼30	24.31 ± 2.93	21.03 ± 6.14	33.76 ± 11.88	38.00 ± 10.74	57.69 ± 26.31	50.34 ± 19.50
	Female	29	20∼30	23.21 ± 2.46	20.28 ± 6.04	32.21 ± 7.38	38.76 ± 9.48	56.34 ± 25.75	51.55 ± 21.22
	All	58	18∼30	23.76 ± 2.74	20.66 ± 6.05	32.98 ± 9.83	38.38 ± 10.05	57.02 ± 25.81	50.95 ± 20.21
Total	Male	57	18∼30	23.65 ± 2.52	21.23 ± 5.78	33.95 ± 11.06	38.72 ± 10.21	58.39 ± 24.62	50.09 ± 19.21
	Female	58	19∼30	23.10 ± 2.54	20.72 ± 6.09	33.09 ± 8.01	38.57 ± 9.19	55.24 ± 25.32	49.22 ± 20.15
	All	115	18∼30	23.37 ± 2.53	20.97 ± 5.92	33.51 ± 9.61	38.64 ± 9.66	56.80 ± 24.92	49.65 ± 19.61

Data are presented as mean ± standard deviation (SD) except for the age range.

Participants reported no abnormal neurological history, had normal or corrected-to-normal vision, and were right-handed. They provided written informed consent prior to participation and were compensated to participate in the study. All study procedures were approved by the Institutional Review Board (2018-IRBH-001).

### 2.2. Stimuli

Before the experiments, images of faces with neutral emotional expressions were taken for each of the four confederates in a controlled environment with constant artificial light at a fixed distance (80 cm) between face and camera. None of the confederates were familiar to the participants. Face images were processed using Adobe Photoshop v21.0.6 to remove hair, ears, and background. Low-level image properties (mean luminance) were equated using the SHINE color toolbox (Willenbockel et al., 2010; Dal Ben, 2019).

### 2.3. Procedures

To evaluate the potential effects of autistic traits and anxiety of participants on PS and, more importantly, to explore the generalization of the modulation effects of social interaction on participants with different degrees of autistic traits and anxiety, we collected Autism Spectrum Quotient (AQ) and State-Trait Anxiety Inventory (STAI) questionnaires in the present study. Before the experiment, each participant completed two self-report questionnaires, i.e., Chinese versions of the AQ (Baron-Cohen et al., 2001) and STAI (Spielberger et al., 1970).

**FIGURE 1:**
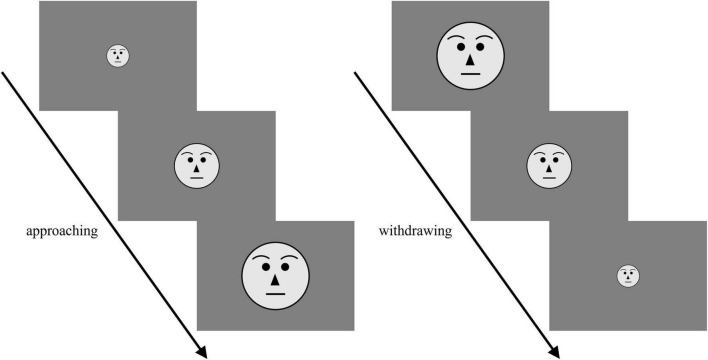
Schematic of computerized stop-distance. Moving face stimuli of four confederates in approaching and withdrawing directions were displayed. Participants pressed the space key to stop the movement of the image at their preferred distance.

Experiments were performed using Presentation software v21.1. Participants were seated in a comfortable chair in a sound-attenuated room. All stimuli were presented against a uniform gray background on a 22-inch monitor (Dell P2217H, 1,920 × 1,080 pixels). To ensure the PS measurement procedures were the same before and after social interaction, we applied an adapted computerized version of the stop-distance paradigm (Figure 1), a validated method for measuring personal space size [see Review in Huang and Izumi (2021)]. When viewed from 64 cm, the face images subtended a visual angle of 3.28° (H) × 3.28° (W) to 15.30° (H) × 15.30° (W), simulating the confederates walking toward the participant from 3 m to 64 cm at a rate equivalent to a speed of 15.73 cm/s, and vice versa. Participants were instructed to press the space key to stop the movement of the image at their preferred distance (i.e., distance at which they felt “most comfortable,” used as a reference to the distance they maintain from a stranger).

Experiments started with a four-trial practice phase, in which a cartoon image was presented. The initial PS (termed pre-PS) measurement experiment was conducted using face images and consisted of 24 trials [4 confederates × 2 directions (approaching or withdrawing) × 3 repetitions]. The order of face images was pseudorandomized to avoid the same confederates appearing in consecutive trials.

Next, participants underwent a 30-min game session. At the beginning of the game session, the rules were introduced to participants in both groups. In this study, each participant of the Cooperative group was randomly assigned a confederate (termed “the cooperative confederate”) of the same sex to minimize variabilities during social interaction. The rest three confederates were treated as non-cooperative confederates. During the social interaction, participants might become more familiar with the cooperative confederate but not the non-cooperative confederates. By comparing PS with cooperative and non-cooperative confederates before and after social interaction, we could disentangle the effect of familiarization from the impact of social interaction. For the Cooperative group, each participant cooperated with the cooperative confederate to play three games (i.e., charades, tossing, and blind drawing) in a fixed order with increasing difficulty. For the Control group, the above three games were played by participants themselves alone in the same room.

In the charades game, words were described by the confederate facing the participant (the Cooperative group) or by the written sentence on the paper (the Control group), with participants then guessing the words. In the tossing game, participants fastened a conical hat in front of their face (with a hole in the top through which they could see out), then collected balls scattered on the floor and tossed them into cups on the other side of the room with or without the help of the confederate. In the blind drawing game, participants first observed obstacles on the floor and a maze on the whiteboard. Then, wearing a sleep mask, they turned round three times, walked through the obstacles, and drew a line from the start to end point of a maze with or without the help of the confederate.

After the game session, PS was again measured for each participant using the same paradigm as before (termed post-PS).

Participants completed a questionnaire after the above sessions, in which they were asked to: (1) evaluate their experience with the game sessions (e.g., whether they understood the game rules), (2) estimate their actual and expected proportion of time spent alone and accompanied by others during their waking hours, and (3) confirm that they were unfamiliar with the confederates before the experiments. The Cooperative group participants were also required to answer whether the cooperator in the game session was one of the confederates in the PS measurement session.

### 2.4. Data analysis

Personal space was converted from the stimulus video frame number (*FN*) when participants pressed a key to choose the distance at which they felt comfortable with the approaching (Eq. 1) or withdrawing faces (Eq. 2).


(1) A⁢p⁢p⁢r⁢o⁢a⁢c⁢h⁢i⁢n⁢g⁢P⁢S=300-(F⁢N-1)×((300-64)/(375-1))

(2) W⁢i⁢t⁢h⁢d⁢r⁢a⁢w⁢i⁢n⁢g⁢P⁢S=64+(F⁢N-1)×((300-64)/(375-1))

where 300 (cm) is the maximum distance between the face and participant, 64 (cm) is the minimum distance between the face and participant, and 375 is the total number of frames between the maximum and minimum distances.

Participants’ PS was averaged for the approaching and withdrawing directions for each confederate, respectively.

To limit the effect of individual differences, the change rate of PS before and after the game session was calculated (Eq. 3).


(3) C⁢h⁢a⁢n⁢g⁢e⁢R⁢a⁢t⁢e=(p⁢o⁢s⁢t-P⁢S-p⁢r⁢e-P⁢S)/p⁢r⁢e-P⁢S

The PS and questionnaire data were analyzed using SPSS v25.0 and GraphPad Prism v8.2.1.

We used one-sample *t*-tests to compare the change rate of PS in different groups with zero to evaluate the moderating effect of social interaction on PS, with adjustment for multiple testing using the Holm-Bonferroni method.

To explore the modulatory differences between the Cooperative and Control groups and the generalization of cooperation on PS, we conducted analysis of variance (ANOVA) with Group (Control and Cooperative) as the between-subject factor, with or without Confederate (Cooperative and Non-cooperative) and/or Sex as the within-subject factors. Note that, in this study, each participant was randomly assigned a confederate of the same sex to minimize variabilities during social interaction. As such, for the Cooperative group, the sex of the participants was the same as the cooperative confederates. Greenhouse–Geisser correction was used for deviations from sphericity when required. We then performed *post-hoc* tests using the Bonferroni method.

We also performed Pearson correlation analyses (two-tailed) to assess the relationship between PS results and questionnaire data.

## 3. Results

### 3.1. The potential factors that may affect PS

Previous studies have demonstrated that PS requirements differ among different age groups (Burgess, 1983; Rapp and Gutzmann, 2000; Webb and Weber, 2003; Gérin-Lajoie et al., 2006; Ozdemir, 2008; Sorokowska et al., 2017). To evaluate the potential influence of age on our results, we conducted the Pearson correlation to assess the potential relationship between participants’ pre-PS and age. A significant positive correlation was found (*r* = 0.185, *p* = 0.048), indicating that the younger the participants, the smaller their preferred PS, in line with previous studies. Note that in the present study, all participants belonged to a relatively narrow age range (18–30 years), corresponding to young adults in previous studies [e.g., 18–29 years in Ozdemir (2008); 22–35 years in Gérin-Lajoie et al. (2006)]. Therefore, we conducted the following analyses with Age as a covariate instead of dividing the participants into different age groups.

We conducted a three-way ANOVA with Direction (approaching and withdrawal) and Confederate (f1, f2, m1, and m2) as within-subject factors, Sex of participants as a between-subject factor, and Age as a covariate on all the participants’ pre-PS. We observed a significant main effect of Direction on pre-PS [*F*(1,112) = 10.849, *p* = 0.001, $\eta_{p}^{2}$ = 0.088], with greater PS found in the approaching condition than in the withdrawal condition, in line with previous research (Vieira et al., 2017). Therefore, we included Direction as a within-subject factor in the following analyses. The main effect of Confederate was not significant [*F*(1.987,222.523) = 0.377, *p* = 0.685, $\eta_{p}^{2}$ = 0.003], indicating that the PS with the four confederates were similar to each other. Therefore, we collapsed the results across four confederates to ensure the robustness of our findings. We also did not find significant main effect of Sex of participants [*F*(1,112) = 0.717; *p* = 0.399; $\eta_{p}^{2}$ = 0.006].

### 3.2. Significant modulatory effects of social interaction on PS in the Cooperative group but not in the Control group

We compared the PS change rates between the two groups using two-way ANOVA with Group (Control and Cooperative) as the between-subject factor and Direction (approaching and withdrawal) as the within-subject factor, with Age as the covariate. Results showed that the PS change rate in the Cooperative group was significantly greater than that in the Control group [*F*(1,112) = 3.964; *p* = 0.049; $\eta_{p}^{2}$ = 0.034], confirming that social interaction rather than single-person tasks or re-tests reduced participant PS, consistent with previous studies (Gessaroli et al., 2013; Patané et al., 2017). No significant interaction effect of Direction with Group was found [*F*(1,112) = 0.590; *p* = 0.444; $\eta_{p}^{2}$ = 0.005], indicating that the results of PS change rates in both directions were similar.

**FIGURE 2:**
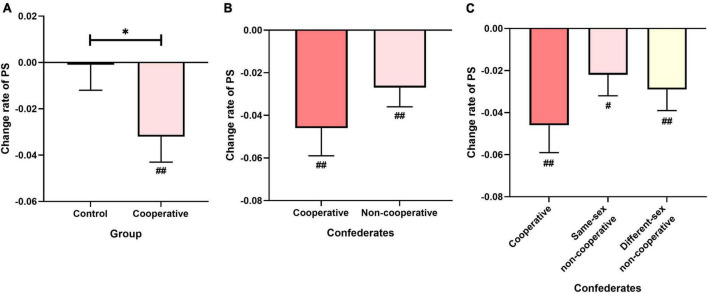
Experimental results of modulatory effects of social interaction on PS. **(A)** Change rate of PS in Control and Cooperative groups. **(B,C)** Change rates of PS in Cooperative group as a function of confederate type. Error bars indicate standard errors of the mean. For ANOVA: *p < 0.05. For one-sample *t*-test (two-tailed): black *^#^p* < 0.05, ^##^*p* < 0.01 Holm-Bonferroni corrected; red *^#^p* < 0.05 uncorrected.

To further explore the effects of social interaction on PS, we also conducted one-sample *t*-tests (two-tailed) to examine whether the PS change rates in the Control and Cooperative groups were significantly different from zero. As shown in Figure 2A, the PS change rate in the Control group did not differ from zero [*t*(56) = 0.067, *p* = 0.947], indicating that PS was not affected by single-person tasks or re-tests. In contrast, the PS change rate in the Cooperative group was significantly less than zero [*t*(57) = 3.682, *p* = 0.001], indicating that PS was significantly reduced after cooperative interaction.

### 3.3. Generalized modulatory effects of social interaction on PS from cooperative to non-cooperative confederates

Next, we examined whether the observed modulatory effects of social interaction on PS in the Cooperative group could be generalized from cooperators to non-cooperators. We divided the Cooperative group into two subgroups based on the role of confederates: cooperative and non-cooperative. The repeated two-way ANOVA with Confederate (cooperative and non-cooperative) and Direction (approaching and withdrawal) as within-subject factors and Age as the covariate showed no significant difference between the PS change rates with the cooperative and non-cooperative confederates [*F*(1,56) = 2.134; *p* = 0.150; $\eta_{p}^{2}$ = 0.037]. Again, no significant interaction effect of Direction with Confederate was found [*F*(1,56) = 3.425; *p* = 0.069; $\eta_{p}^{2}$ = 0.058]. Furthermore, one-sample *t*-tests (two-tailed) revealed that the PS change rates in both cooperative [*t*(57) = 3.366; *p* = 0.003] and non-cooperative confederates [*t*(57) = 3.110; *p* = 0.003] were significantly less than zero, indicating that cooperative interaction reduced PS not only with cooperators but also with non-cooperators (Figure 2B).

To further investigate whether the generalization effects also exist among confederates by sex, that is, whether the modulatory effects of social interaction on PS could generalize to the non-cooperative confederates with the different sex from the cooperative confederates (participants), we further divided non-cooperative confederates into two subgroups: same-sex non-cooperative confederates and different-sex non-cooperative confederates. The repeated two-way ANOVA revealed no significant differences among the three subgroups (cooperative confederates, same-sex non-cooperative confederates, different-sex non-cooperative confederates) sub-groups [*F*(1.57,88.03) = 1.870, *p* = 0.168, $\eta_{p}^{2}$ = 0.032], suggesting that the effect of social interaction on PS with non-cooperative confederates of different sex was similar to that with cooperative confederates. There was no significant interaction effect of Direction with Subgroup [*F*(2,112) = 2.631, *p* = 0.076, $\eta_{p}^{2}$ = 0.045]. In both non-cooperative subgroups, we found significant changes in PS after social interaction using one-sample *t*-tests (two-tailed) [same-sex non-cooperative confederates: *t*(57) = 2.225, *p* = 0.030; different-sex non-cooperative confederates: *t*(57) = 3.189, *p* = 0.005] (Figure 2C), indicating that the effect of cooperative interaction on PS could be generalized across different sex non-cooperative confederates.

### 3.4. Effect of participant sex on social interaction modulation of PS

**FIGURE 3:**
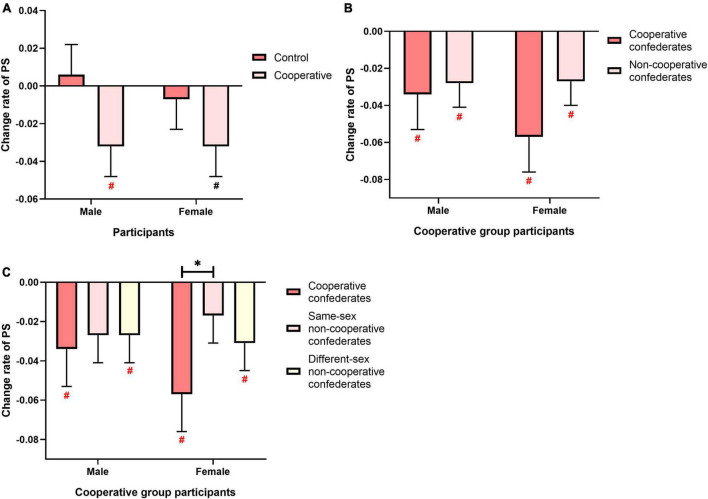
Modulatory effect of social interaction on PS and its generalization in male and female participants. **(A)** Change rate of PS in Control and Cooperative groups. **(B,C)** Change rates of PS in Cooperative group as a function of confederate type. Error bars indicate standard errors of the mean. For ANOVA: *p < 0.05. For one-sample *t*-test (two-tailed): black ^#^*p* < 0.05 Holm-Bonferroni corrected; red ^#^*p* < 0.05 uncorrected.

To further explore potential sex differences in the above findings, we used Sex as a between-subject factor by dividing participants into males and females, then repeated the above analyses (see Figure 3). We conducted a three-way ANOVA with Group and Sex of participants as between-subject factors and Direction as the within-subject factor, with Age as the covariate. Significant main effect of Group [*F*(1,110) = 3.928, *p* = 0.050, $\eta_{p}^{2}$ = 0.034] but not main effect of Sex of participants [*F*(1,110) = 0.173, *p* = 0.678, $\eta_{p}^{2}$ = 0.002] or main effect of Direction [*F*(1,110) = 0.799, *p* = 0.373, $\eta_{p}^{2}$ = 0.007] or interactions among these factors [between Group and Sex of participants: *F*(1,110) = 0.144, *p* = 0.705, $\eta_{p}^{2}$ = 0.001; between Group and Direction: *F*(1,110) = 0.545, *p* = 0.462, $\eta_{p}^{2}$ = 0.005; among Group, Sex of participants and Direction: *F*(1,110) = 1.636, *p* = 0.204, $\eta_{p}^{2}$ = 0.015] was found, indicating that the effects of social interaction on PS were similar between male and female participants in both directions. In addition, one-sample *t*-tests (two-tailed) revealed that the PS change rates in males and females of the Control group did not significantly differ from zero [male: *t*(27) = 0.496, *p* = 0.624; female: *t*(28) = 0.535, *p* = 1.000], whereas the change rates in males and females of the Cooperative group was significantly or near significantly lower than zero [male: *t*(28) = 2.374, *p* = 0.074; female: *t*(28) = 2.835, *p* = 0.034] (Figure 3A).

The generalization effects were also separately tested in male and female participants in the Cooperative group. Again, no significant main effect of Sex of participants (equal to Sex of cooperative confederates) on the generalization was found (Figures 3B, C).

### 3.5. Questionnaire results and correlation analyses

Degree of autism, anxiety, and daily social activities of the participants were calculated (Table 1). We conducted one-way ANOVA and did not find significant differences between the Control and Cooperative groups [AQ: *F*(1,113) = 0.34, *p* = 0.562, $\eta_{p}^{2}$ = 0.003; STAI-state: *F*(1,113) = 0.35, *p* = 0.553, $\eta_{p}^{2}$ = 0.003; STAI-trait: *F*(1,113) = 0.09, *p* = 0.769, $\eta_{p}^{2}$ = 0.001; Actual-Accompanied: *F*(1,113) = 0.01, *p* = 0.925, $\eta_{p}^{2}$ < 0.001; Expected-Accompanied: *F*(1,113) = 0.51, *p* = 0.477, $\eta_{p}^{2}$ = 0.004] or between male and female participants [AQ: *F*(1,113) = 0.21, *p* = 0.650, $\eta_{p}^{2}$ = 0.002; STAI-state: *F*(1,113) = 0.23, *p* = 0.633, $\eta_{p}^{2}$ = 0.002; STAI-trait: *F*(1,113) = 0.01, *p* = 0.934, $\eta_{p}^{2}$ < 0.001; Actual-Accompanied: *F*(1,113) = 0.46, *p* = 0.501, $\eta_{p}^{2}$ = 0.004; Expected-Accompanied: *F*(1,113) = 0.06, *p* = 0.815, $\eta_{p}^{2}$ < 0.001]. We also conducted Pearson correlation analyses with controlling for Age to assess relationships of the pre-PS and change rate of PS with questionnaire results, with no significant correlations found (all *p* > 0.05).

## 4. Discussion

In the present study, we investigated whether the modulatory effect of social interaction on PS is simply due to familiarization with confederates, and if not, whether it can be generalized to confederates with whom participants do not interact. Our findings confirmed that PS can be modulated by social interaction, consistent with previous studies. Importantly, this modulatory effect was not simply due to confederate familiarization but was indeed due to social interaction. Furthermore, we found that the impact of social interaction on PS could be generalized to non-cooperative confederates, even those of a different sex than cooperative confederates.

We conducted three cooperative tasks and measured participant PS before and after to investigate the effect of social interaction on PS. We found that PS of participants in the Cooperative group decreased after social interaction. Several possible scenarios may account for this observed decrease: i.e., (1) time interval between the first and second PS measurement, (2) familiarization with the PS measurement task, (3) familiarization with confederates, and (4) social interaction. We did not find similar results in the Control group, which used the same time intervals and tasks between PS measurements. Therefore, the first and second scenarios are unlikely. Previous studies have reported that familiarity between interacting parties may affect PS (Little, 1965; Willis, 1966; Cristani et al., 2011). However, this familiarity (third scenario) did not explain our results. In contrast to one confederate used in previous studies, we enrolled four confederates and selected one as the cooperative confederate. Thus, after social interaction, PS was measured not only with cooperative confederates but also with non-cooperative confederates. Indeed, PS was reduced for all confederates, including non-cooperators with whom participants did not become familiar during the social interaction session. Our findings provide clear evidence for the modulatory effects of social interaction on PS, consistent with previous research on the effect of observing other’s social interactions on interpersonal distance of a third-person perspective. For example, third-person perspective of interpersonal distance can be influenced by watching a child and a female adult building Legos in a cooperative or uncooperative manner and observing someone intentionally or unintentionally helping or harming others (Candini et al., 2017; Shao et al., 2020). Note that PS change rate in the Cooperative group was not correlated with individual characteristics (e.g., degree of autism and anxiety), indicating that the modulatory effect of social interaction on PS may exist in a wide range of populations.

The regulatory effect of social interaction on PS may be due to the influence of many psychological factors induced by social interaction, such as trust (King-Casas et al., 2005; Fett et al., 2014; Rosenberger et al., 2020; Massaccesi et al., 2021) and anxiety (Rinck et al., 2010; Wieser et al., 2010). Unlike tasks used in previous studies [e.g., reading together (Gessaroli et al., 2013) and playing Lego together (Candini et al., 2017)], the three cooperative tasks used in the present study were designed to increase participant trust in the cooperative confederate from tasks 1 to 3. It has been shown that post-game PS is associated with experienced trust during the game. For example, interactions with a selfish trustee increase participant PS, while interactions with a cooperative trustee decrease participant PS (Rosenberger et al., 2020; Massaccesi et al., 2021). Furthermore, anxiety may also affect individual PS. Previous research has shown that those receiving peer ratings for social competence and sexual attractiveness, and thus experience stress, exhibit greater PS than controls (Dosey and Meisels, 1969). Rinck et al. (2010) also reported that participants with higher anxiety levels show a slower pace and greater PS when approaching an avatar in an immersive virtual reality.

A key finding of this study is that the modulatory effect of social interaction on PS generalized to confederates with whom participants did not interact. Previous studies only measured PS before and after social interaction with one confederate (Gessaroli et al., 2013; Candini et al., 2017, 2019; Patané et al., 2017; Rosenberger et al., 2020; Massaccesi et al., 2021), and thus could not determine whether the modulatory effect was generalizable. We overcame this limitation by using four confederates (two females and two males), only one of which was treated as a cooperative confederate for each participant. We found that after half an hour of social interaction, PS was reduced regardless of confederate type (cooperative or non-cooperative). Even PS with non-cooperative confederates of a different sex than the cooperative confederate was also reduced after social interaction. Previous studies have found that PS can be regulated by long-term interactions, e.g., infant attachment to a caregiver (Cassidy and Berlin, 1994; Bar-Haim et al., 2002). Bar-Haim et al. (2002) found that the PS of children is significantly related to attachment security/insecurity with caregivers (e.g., mothers and professional caregivers) during infancy. Our study suggests that transitory social interactions can also regulate general PS, not just PS with specific cooperative confederates. This generalization of the modulatory effects of social interaction on PS is consistent with previous studies in ASD groups showing successful generalization of the improved effects of empathic interventions and social-communication across materials and populations (Argott et al., 2017; Hong et al., 2018; Carruthers et al., 2020). For example, modeling, prompting, and reinforcement can improve the ability of children with ASD to differentiate between types of affective stimuli and generate complex empathic responses, and this improvement can even be generalized to untaught stimuli and novel adults (Argott et al., 2017). Several recent reviews have also shown successful generalizations cross people, setting, activities, materials, and behaviors in studies on social communication interventions for ASD groups (Hong et al., 2018; Carruthers et al., 2020).

## 5. Conclusion

In summary, our findings provide clear evidence for the meditating role of social interaction on PS. Importantly, this modulatory effect can be generalized to confederates with whom participants do not interact. Thus, proper social interaction may affect general PS, not just PS with one specific individual. These results should help deepen our understanding of the regulation of PS. Moreover, our results may have implications beyond the healthy population studied in the present study and benefit people with dysfunctional social behaviors. For example, individuals with ASD often have abnormal PS requirements and regulations compared to typically developing individuals. Our findings on the modulatory effect of social interaction on PS may have important implications for developing interventions and therapies for children with ASD.

## Data availability statement

The raw data supporting the conclusions of this article will be made available by the authors, without undue reservation.

## Ethics statement

The studies involving human participants were reviewed and approved by the Institutional Review Board, Institute of Biophysics, CAS. The patients/participants provided their written informed consent to participate in this study.

## Author contributions

SC: conceptualization, methodology, validation, data collection, investigation, formal analysis, writing–original draft, and writing–review, and editing. TY: conceptualization and methodology. NL: conceptualization, methodology, writing–original draft, writing–review and editing, supervision, and project administration. All authors contributed to the article and approved the submitted version.
